# TACE-assisted multi-image guided radiofrequency ablation for the treatment of single hepatocellular carcinoma ≤ 5 cm: a retrospective study

**DOI:** 10.3389/fonc.2024.1347675

**Published:** 2024-04-05

**Authors:** Yong Xie, Tianshi Lyu, Li Song, Xiaoqiang Tong, Jian Wang, Yinghua Zou

**Affiliations:** Department of Interventional Radiology and Vascular Surgery, Peking University First Hospital, Beijing, China

**Keywords:** hepatocellular carcinoma (HCC), transarterial chemoembolization (TACE), radiofrequency ablation (RFA), local tumor progression (LTP), overall survival (OS), complication

## Abstract

**Background/Objective:**

Treatment of hepatocellular carcinoma (HCC) with ablation alone often results in high rates of recurrence and metastasis, reaching up to 25.9% within two years. Therefore, this study aimed to examine the efficacy and safety of transarterial chemoembolization (TACE)-assisted multi-image guided radiofrequency ablation (RFA) for the treatment of stage Ia HCC according to the China liver cancer staging (CNLC).

**Methods:**

This study enrolled and analyzed a total of 118 patients diagnosed with HCC, each with a single nodular lesion no larger than 5 cm, who received TACE-RFA as first-line therapy between February 1, 2014, and December 31, 2021. The median/mean follow-up period was 29.0 months [95% confidence interval (CI): 21.8-36.2 months] and 31.8 months (95% CI: 27.5-36.0 months), respectively. We assessed the treatment’s effectiveness, potential complications, and survival rate.

**Results:**

The technical success rate was 100% (118/118) after the initial treatment. Out of the total, 3 out of 118 patients (2.5%) developed local tumor progression (LTP) during the follow-up period. The median time for LTP was 29.0 months (95%CI: 21.9-36.1 months; mean: 31.5 months; range 1-92 months). At 1, 3, 5, and 7 years after treatment, the cumulative LTP rates were 0%, 4.6%, 4.6%, and 4.6%, respectively. The overall survival rates at 1, 3, 5, and 7 years were 100%, 95.2%, 95.2%, and 95.2%, respectively. In total, 28 patients experienced minor Grade B complications, and no major complications or treatment-related mortality occurred.

**Conclusion:**

The treatment of CNLC stage Ia HCC using TACE-assisted multi-image-guided RFA was found to be both safe and feasible.

## Introduction

Hepatocellular carcinoma (HCC) is one of the most common malignancies worldwide (1). According to recent research (2), an estimated 905,700 new liver cancer diagnoses are expected globally in 2020, leading to 830,200 deaths from the disease. China accounts for half of the world’s new cases and deaths from liver cancer.

HCC accounts for approximately 75-85% of liver cancer cases. The differences in pathogenesis, biological behavior, histopathology, treatment, and prognosis of different types of liver cancer contribute significantly to the global healthcare burden. HCC is a prevalent subtype of liver cancer and a malignant tumor of the digestive tract, characterized by a high degree of malignancy, disease progression, mortality rate, and poor efficacy. In most cases, HCC presents with no typical clinical signs in its early stages, and nearly 70-80% of patients are diagnosed at advanced stages, resulting in a missed opportunity for optimal surgical intervention (3).

Radiofrequency ablation (RFA) remains the primary treatment for minimally invasive management of unresectable early-stage HCC. Transarterial chemoembolization (TACE) is recommended as the first-line treatment for intermediate-stage HCC. However, despite its widespread use, the long-term efficacy of RFA is unsatisfactory due to suboptimal ablation boundaries (4). This results in estimated 3- and 5-year survival rates of 60-84% and 40-68%, respectively (5). Additionally, conventional ultrasound (US)-guided RFA is associated with a high rate of tumor recurrence, particularly when ablating poorly developed lesions or difficult-to-locate tumors, such as those located near the diaphragm (6, 7). Recent studies using iodide oil (4, 6) to label tumor lesions through the arterial route have shown significant improvement in long-term survival prognosis and satisfactory, clear, safe ablation boundaries. However, the safety and efficacy of TACE-assisted multi-image-guided RFA for the treatment of stage Ia HCC (i.e., performance status score 0~2, Child-Pugh grade A/B liver function, single tumor ≤5cm, no vascular invasion and extrahepatic metastasis) based on the China liver cancer staging (CNLC) (8) is unclear.

Therefore, we aimed to investigate the clinical safety and efficacy of TACE-assisted multi-image-guided RFA for the treatment of stage Ia HCC in patients with CNLC using ultrasound, computed tomography (CT), or cone beam CT to leverage their respective strengths and advantages, minimize the risk of local recurrence, and enhance the safety zones of ablation.

## Materials and methods

### Patients

A retrospective cohort study was carried out on patients diagnosed with HCC who underwent TACE-RFA as their initial treatment at our institution from February 1, 2014, to December 31, 2021. The study was approved by the institutional review board, and written informed consent was waived due to the retrospective nature of the analysis.

The study’s inclusion criteria comprised patients with the following characteristics: (a) histopathologically confirmed diagnosis of HCC or diagnoses compliant with the guidelines of the European Association for the Study of Liver/American Association for the Study of Liver Disease (9); (b) diagnosed with stage Ia HCC; (c) aged 18 years or older; (d) classified as Child-Pugh Grade A or B; (e) Eastern Cooperative Oncology Group performance status (ECOG PS) of 0-1; (f) patients who had declined surgery. Exclusion criteria encompassed: (a) pregnancy, lactation, or of childbearing age; (b) acute infection or acute onset of chronic infection; (c) patients with hepatitis B virus (HBV) or hepatitis C virus (HCV) without regular antiviral therapy; (d) presence of malignant tumors in sites other than the liver; (e) prior radiotherapy, chemotherapy, molecular targeting, or immunotherapy; (f) loss to follow-up; (g) incomplete imaging data; (h) microwave or cryoablation following TACE; (i) TACE or RFA as monotherapy; (j) RFA followed by TACE. Patients meeting the inclusion criteria were enrolled and analyzed.

### TACE procedure

Under the guidance of digital subtraction angiography (DSA), the 5-Fr Rosch hepatic catheter (Cook, Bloomington, IN, USA) was routinely chosen for intubating the hepatic artery or superior mesenteric artery to assess the tumor blood supply. Subsequently, a 1.9-French microcatheter (Asahi Intecc Co., Ltd., Japan) was employed to super selectively access the tumor’s feeding arteries. Cone beam computed tomography (CBCT) with dynamic contrast and three-dimensional reconstruction was performed using the microcatheter, and the blood supply to the lesion was identified at the post-processing station. Additionally, careful attention was given to detecting any abnormal tumor-stained nodules, and microcatheters were utilized for embolization whenever possible. A mixture of 5-10 mL of iodide oil (Guerbet, Villepinte, Seine-Saint-Denis, France) and 10-30 mg of epirubicin was emulsified in specific proportions and used to embolize the feeding arteries. Lesions with abundant blood supply were embolized with gelatin sponge granules or embolic microspheres in the interim, and the embolization was ceased when blood flow was essentially stagnant. Finally, the iodized oil deposition of the tumor was checked through three-dimensional reconstruction of CBCT, and embolization was performed as needed until satisfactory embolization was achieved.

### RFA procedure

**Figure 1:**
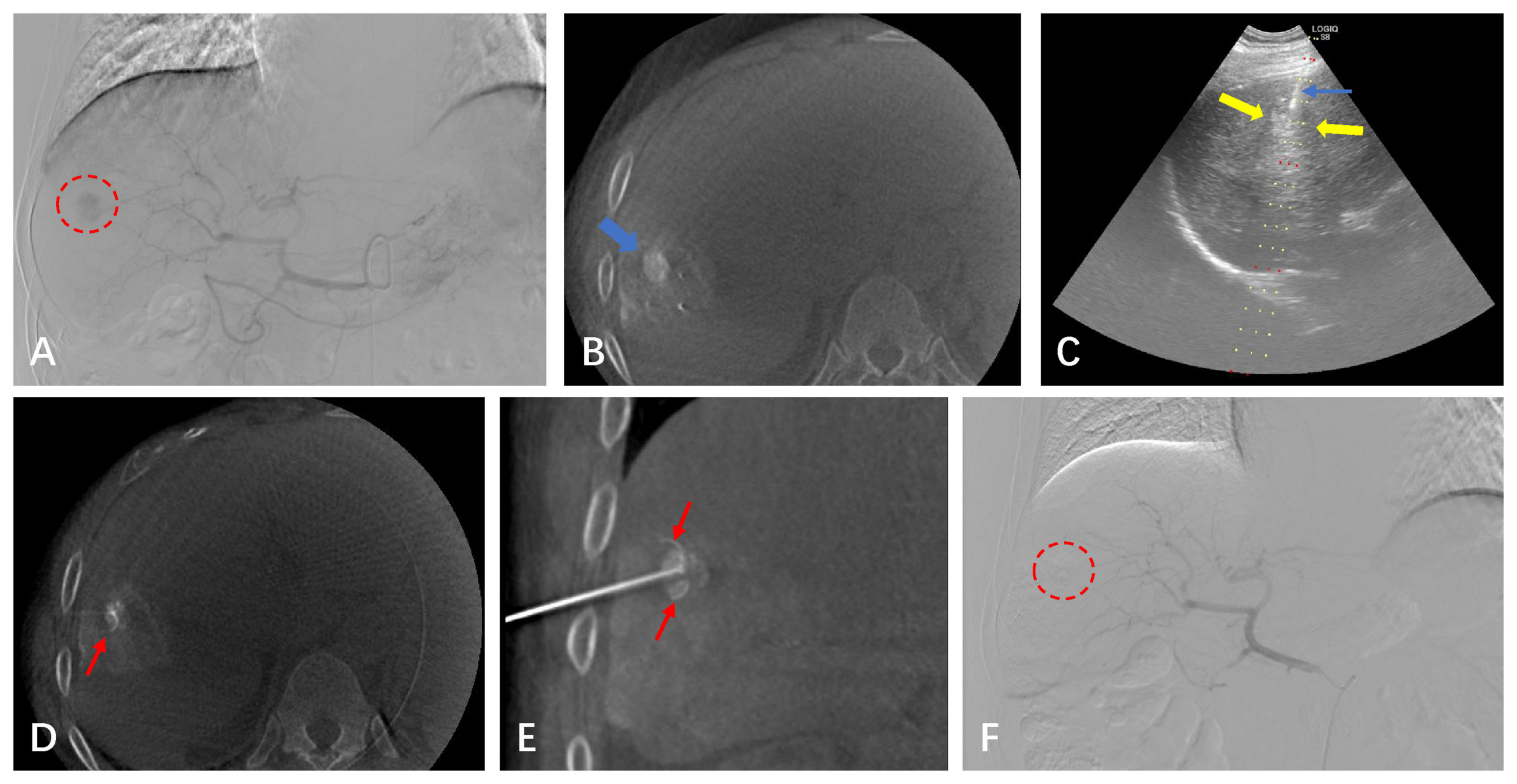
A 64-year-old male with a small HCC of 1.8 cm in the right hepatic lobe. **(A)** CBCT-guided angiography shows the HCC lesion (red circle). **(B)** After undergoing the TACE procedure, the CBCT scan shows dense iodinated oil deposits in the lesion (blue arrow). **(C)** Real-time puncture under fluoroscopy with US guidance. **(D, E)** CBCT image confirming the position of the RFA needle (red arrow). **(F)** After the lesion underwent simultaneous TACE-RFA, CBCT-guided selective angiography does not show the original abnormal staining of the small HCC.

We conducted RFA procedures within 4 weeks following TACE. The median time interval between TACE and RFA was 4 days, with a range of 0-30 days. The RFA procedure was guided by a combination of imaging equipment, including ultrasound combined with CT or ultrasound combined with CBCT, and DSA if needed. Ultrasound was primarily used for real-time monitoring of tumor puncture and ablation processes, while CT/CBCT was used to assess ablation areas and safety boundaries. DSA was utilized for fluoroscopic adjustment of multipolar ablation needles in cases of obvious lesions with iodide oil deposits (**Figure 1**). Depending on the manufacturer’s power settings, we employed the Boston Medical Device (RF 3000TM, Boston Scientific Way, Marlborough, MA 01752, US). The choice of electrode needle (LeVeenTMSuperSlimTM, Needle Electrode System, Boston Scientific) often depended on the size of the tumor and the location of the lesion.

First, the positioning of ultrasound is combined with preoperative CT and magnetic resonance imaging (MRI) to select the appropriate puncture point and needle angle. After administering local anesthesia with 1% lidocaine, the patient was instructed to hold their breath, and the multipole needle was inserted subcutaneously close to the liver envelope. Then, the patient was again instructed to hold their breath, and the multipole needle was inserted into the target lesion. The direction of the puncture needle was fine-tuned under ultrasound scan until the multipole needle fully covered the lesion. Real-time ultrasound was used to monitor tumor ablation progression. This was followed by multiple CT/CBCT scans to determine if there was a sufficient ablation area (greater than 5-10 mm). If not, the position of the needle tip was adjusted several times. Finally, the puncture channel was ablated during electrode retraction to prevent bleeding or tumor cell seeding.

All patients underwent repeat abdominal contrast MRI or CT within 1 month after initial RFA to assess for residual lesions after ablation. Immediate remedial RFA was conducted if the lesion was still viable. All procedures are performed by the same team at our center, with more than 10 years of experience in interventional therapy.

### Variable collection and follow-up strategies

The general information collected includes age, sex, alcohol consumption, smoking status, presence of cirrhosis of the liver, tumor location, tumor maximum diameter, tumor burden score (TBS) (10), Child-Pugh score, type of liver disease, ECOG PS, relevant laboratory tests within 7 days before treatment, and so on. The primary endpoints were local tumor progression (LTP), and the secondary endpoints were technical success, overall survival (OS), and postoperative complications. LTP is defined as the presence of a newly enhanced tumor within the original ablation zone. Technical success rate is defined as a repeat abdominal contrast MRI or CT within 1 month after RFA to assess the absence of tumor remnants in the ablation area. OS is defined as the time from the start of RFA treatment to death from any cause. Complications include local hematoma, bleeding, local skin infection, pain, gastrointestinal reactions (e.g., nausea and vomiting), and so on, classified with reference to the Society Guidelines for Interventional Radiology (11), and major complications are defined as any adverse event leading to additional treatment, such as an increased level of care, prolonged hospital stay, fatality, or disability. Intrahepatic distant recurrence (IDR) was defined as any new emerging tumor that occurred in the liver separate from the ablated zone. Extrahepatic recurrence was defined as the appearance of new lesions outside the liver postoperatively. Portal vein tumor thrombus (PVTT) is defined as a lesion in the portal vein, observable in all stages of CT/MRI, with partial enhancement in the arterial phase and a filling defect in the portal vein phase. Liver tumor progression, including LTP, PVTT, IDR, and extrahepatic recurrence, was measured from the time of the RFA procedure to either local or distant progression or death, whichever came first. Patients were followed up with outpatient or inpatient visits every 3 months for the first year after treatment and every 3-6 months thereafter to assess clinical symptoms, conduct relevant laboratory tests, and undergo contrast-enhanced CT scans or MRI examinations. Follow-up time, tumor progression, and patient survival were recorded. If tumor progression is found during follow-up, the best treatment strategy will be carried out according to the latest liver cancer diagnosis and treatment guidelines based on individual conditions (including ablation, surgical resection, liver transplantation, TACE, systemic therapy, radiation therapy, or combination therapy).

### Statistical analysis

Data analysis was performed using R statistical software (version 3.6.1). Measurement data with a normal distribution were reported as mean ± standard deviation. Pairwise t-tests or ANOVA were utilized for group comparisons. Counting data was presented as a percentage, and group comparisons were assessed using the chi-square test. The Kaplan-Meier method was employed to calculate LTP and OS, and group differences were evaluated using the Log-rank test. A P-value of less than 0.05 (two-sided) was considered statistically significant.

## Results

### Baseline characteristics of the patients

A total of 118 patients were included in this study, of whom 92 (77.97%) were male and 26 (22.03%) were female, with a mean age of 59.47 ± 11.82 (range, 29.00-89.00) years. The maximum tumor diameter was 2.32 ± 1.03 (range, 0.70-5.00) cm, and the tumor burden score was 2.56 ± 0.95 (range, 1.22-5.10). Among the included patients, 31 (26.27%) had tumors located in the left lobe of the liver, while 87 (73.73%) had tumors located in the right lobe of the liver. Twenty-one (17.80%) cases had diabetes, 32 (27.12%) had hypertension, 52 (44.07%) were smokers, and 4 (3.39%) were alcohol consumers. With respect to hepatitis, 93 (78.81%) cases involved hepatitis B or C, 4 (3.39%) involved alcoholic or autoimmune hepatitis, and 21 (17.80%) were normal. Furthermore, 109 (92.37%) cases had an ECOG PS of 0, while 9 (7.63%) had an ECOG PS of 1. The incidence of liver cirrhosis was 75 (63.56%), and 54 (46.15%) were in albumin bilirubin (ALBI) grade 1, while 63 (53.85%) were in ALBI grade 2. Additionally, 102 (88.70%) were in Child-Pugh grade A, while 13 (11.30%) were in grade B (**Table 1**).

**Table 1:** Basic characteristics of 118 patients who received TACE-RFA as the first-line treatment for HCC.

Variables	Value
Age, years^§^	59.47 (11.82)/57.00 (29.00-89.00)
Sex, n (%)	
Female	26 (22.03)
Male	92 (77.97)
TBS^§^	2.56 (0.95)/2.24 (1.22-5.10)
Maximum tumor diameter, cm^§^	2.32 (1.03)/2.00 (0.70-5.00)
Maximum tumor diameter, n (%)	
<3	89 (75.42)
≥3, ≤5	29 (24.58)
ALBI score ^†§^	-2.53 (-0.45)/-2.57 (-1.43–3.48)
ALBI, n (%) ^†§^	
Grade 1	54 (46.15)
Grade 2	63 (53.85)
Hypertension, n (%)	
No	86 (72.88)
Yes	32 (27.12)
Diabetes, n (%)	
No	97 (82.20)
Yes	21 (17.80)
Smoking, n (%)	
No	66 (55.93)
Yes	52 (44.07)
Alcohol abuse, n (%)	
No	84 (71.19)
Yes	34 (28.81)
Is TACE performed concurrently with RFA, n (%)	
No	96 (81.36)
Yes	22 (18.64)
Location of the tumor, n (%)	
Left	31 (26.27)
Right	87 (73.73)
BCLC stage, n (%)	
0	61 (51.69)
A	57 (48.31)
Child-Pugh class, n (%)†	
A	102 (88.70)
B	13 (11.30)
Cirrhosis, n (%)	
No	43 (36.44)
Yes	75 (63.56)
ECOG PS, n (%)	
0	109 (92.37)
1	9 (7.63)
Liver disease type, n (%)	
Non-hepatitis	21 (17.80)
Hepatitis B/C	93 (78.81)
Other hepatitis	4 (3.39)
AFP, n (%)	
≤20 ng/ml	78 (66.10)
>20 ng/ml	40 (33.90)
Hemoglobin, g/L^§^	137.09 (17.92)/138.00 (78.00-178.00)
WBC count, 10^9^/L^§^	4.98 (1.91)/4.90 (1.40-12.70)
Platelet count, 10^9^/L^§^	125.70 (68.69)/120.00 (28.00-628.00)
ALT, IU/L^§^	26.45 (18.44)/21.00 (7.00-96.00)
AST, IU/L^§^	32.51 (19.55)/26.00 (12.00-131.00)
Serum albumin, g/L^§^	39.05 (4.91)/39.50 (26.80-50.80)
Total bilirubin, umol/L^§^	17.59 (8.10)/15.70 (4.50-46.00)
Alkaline phosphatase, IU/L^§^	80.32 (31.28)/74.00 (31.00-284.00)
Creatinine, mg/dL^§^	78.13 (14.05)/78.80 (46.20-117.44)
International standardized ratio^§^	1.11 (0.14)/1.09 (0.89-1.60)
Prothrombin time, s^§^	12.20 (1.48)/11.80 (9.30-16.90)

^†^Missing data.

^§^Mean (SD)/Median (range).

TBS, tumor burden score (TBS^2^ = (maximum tumor diameter)^2^ + (number of tumors)^2^); ALBI, albumin bilirubin (ALBI score = (0.66×log10 bilirubin (µmol/L) -(0.085×albumin (g/L))); BCLC, Barcelona Clinic Liver Cancer; ECOG PS, Eastern Cooperative Oncology Group Performance Status; WBC, white blood cell; ALT, alanine transaminase; AST, aspartate aminotransferase.

### Local tumor control rate

**Figure 2:**
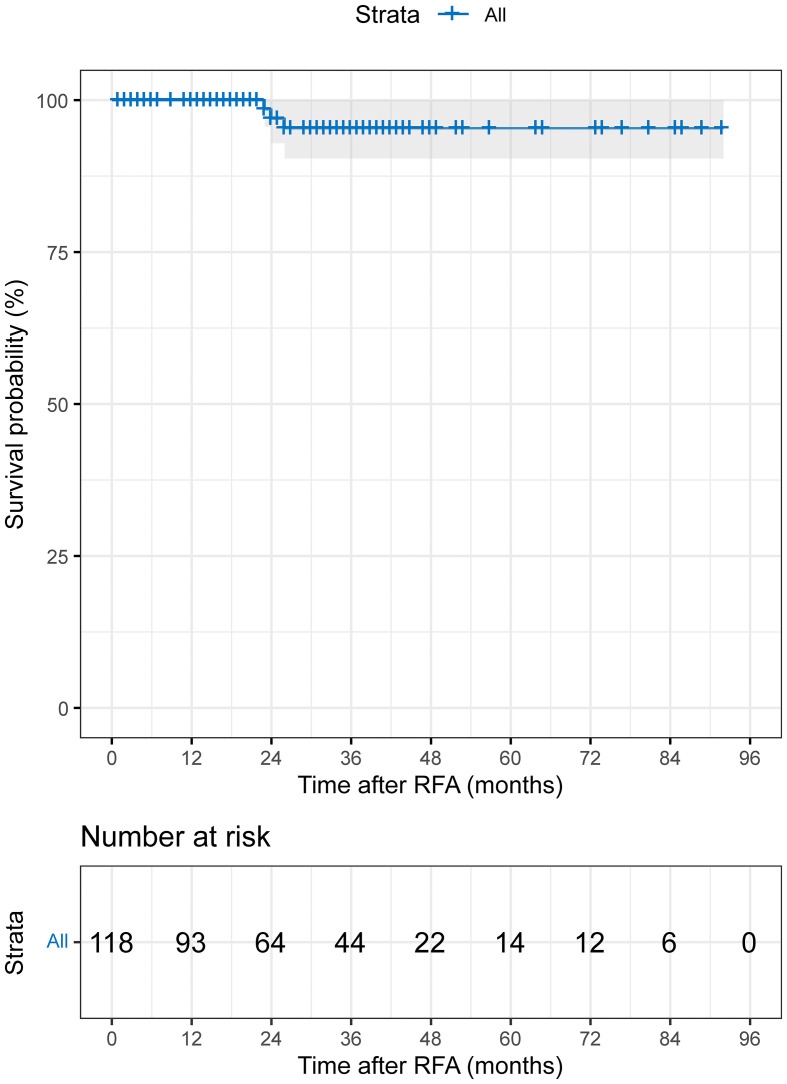
The Kaplan–Meier curve demonstrates the LTP of patients with HCC who received TACE-RFA.

LTP was identified in 3 out of 118 (2.5%) patients during the follow-up period. The median time to LTP was 29.0 months (95% CI: 21.9-36.1 months; range 1-92 months; mean: 31.5 months). The cumulative 1-, 3-, 5-, and 7-year LTP rates were 0%, 4.6%, 4.6%, and 4.6%, respectively (**Figure 2**). In terms of recurrence patterns, 42 cases (35.6%) had IDR, while 3 and 4 cases showed extrahepatic recurrence and PVTT, respectively. Finally, a total of 66 (55.9%) patients did not experience disease progression or recurrence during follow-up (**Table 2**). For disease progression or recurrent lesions, second-line therapy was received, including TACE-RFA (n=23), TACE/TAI (n=15), RFA (n=2), TACE-RFA+systemic therapy (lenvatinib) (n=1), systemic therapy (sorafenib) (n=1), optimal supportive therapy (n=4), and unknown (n=4).

**Table 2:** Events during the follow-up.

Category	Number of patients
Complications¶	
Minor complications	
Grade A	n=80
Grade B	pain (n=26), fever (n=1), vomit (n=1)
Major complications	n=0
Tumor events after RFA	
LTP	n=3
IDR	n=42
Extrahepatic recurrence	n=4
PVTT	n=3
No	n=66
Second-line treatments	
TACE-RFA	n=23
TACE/TAI	n=15
RFA	n=2
TACE-RFA+Systemic therapy	n=1
Systemic therapy	n=1
Support therapy	n=4
Unknown	n=4
No	n=68

¶ Minor complications include grade A and B. Grade A, no therapy, no consequences; Grade B, nominal therapy, no consequence, includes overnight admission for observation only. Major complications include grades C, D, E and F. Grade C, requires therapy, minor hospitalization (< 48 h); Grade D, requires major therapy, unplanned increase in level of care, prolonged hospitalization (> 48 h); Grade E, permanent adverse sequelae; Grade F: death.

LTP, local tumor progression; IDR, intrahepatic distant recurrence; PVTT, portal vein tumor thrombus.

### Overall survival

**Figure 3:**
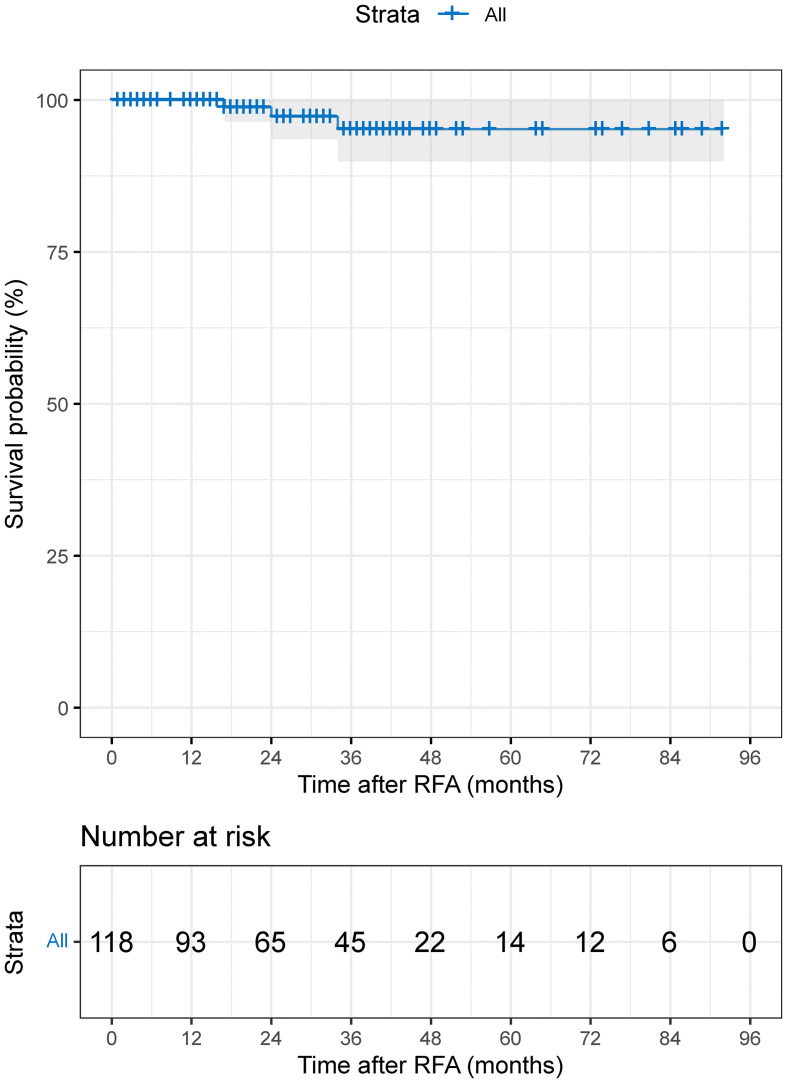
The Kaplan–Meier curve demonstrates the OS of patients with HCC who received TACE-RFA.

The median follow-up period was 29.0 months (95%CI: 21.8-36.2 months; range 1–92 months; mean: 31.8 months). The cumulative 1-, 3-, 5-, and 7-year OS rates were 100%, 95.2%, 95.2%, and 95.2% (**Figure 3**).

### Technical success and complications

After the initial treatment, the technical success rate was 100% (118 out of 118). Twenty-eight patients (23.7%) developed minor grade B complications, which included twenty-six cases of pain in the liver area, one case of fever, and one case of vomiting (**Table 2**). All complications were successfully treated. The major complication rate was 0%. Overall, there were no treatment-related deaths during hospitalization.

## Discussion

Despite constant updates and revisions to the guidelines for diagnosing and treating HCC, the recommended radical treatment strategy for patients with CNLC stage Ia or BCLC stage 0-A HCC has remained unchanged. The challenge, however, is that the survival prognosis for patients at this stage has essentially plateaued, making further improvement difficult. One primary limitation of thermal ablation, a radical treatment method, is that when the tumor diameter exceeds 3 cm, local tumor control diminishes rapidly. Achieving a sufficient range of safe ablation boundary is necessary to control the *in-situ* progression of the local tumor (12). The “heat sink effect,” which results in the dissipation of heat energy, is a key factor affecting the safety boundary. To address this issue, the combination of TACE and thermal ablation has been widely utilized, emerging as a research hotspot in recent years. Our center has achieved a satisfactory rate of tumor local control and survival prognosis using TACE-assisted multi-image-guided RFA, and the procedure has been deemed safe.

TACE followed by RFA has become increasingly popular in recent years, and current clinical data suggest that this combination is superior to using RFA alone. Specifically, TACE in conjunction with RFA induces higher rates of complete necrosis and improves OS. Our research showed that the cumulative 1-, 3-, 5-, and 7-year OS rates were 100%, 95.2%, 95.2%, and 95.2%. Cao et al. (13) conducted several studies on single small HCC (≤ 3.0 cm), and their related studies confirmed that the 1-year, 3-year, and 5-year OS rates of the TACE-RFA and RFA groups were similar (98.7%, 93.0%, and 75.9% *vs*. 97.4%, 88.0%, and 77.4%, P=0.444). In terms of long-term survival benefit (greater than 5 years), an article in the JAMA sub-journal, through a secondary analysis of a phase III randomized controlled clinical study, found that the 5- and 7-year OS rates in the TACE-RFA group versus the RFA group were 52.0% and 36.4% *vs*. 43.2% and 19.4%, respectively (HR=0.55, 95% CI: 0.39-0.78, P=0.001). Subgroup analysis also confirmed that the OS of the TACE-RFA group was significantly better than that of the RFA group (HR=3.20, 95% CI=1.91-5.35, P<0.001) regardless of the diameter (>3 cm *vs*. ≤3 cm) (14). Yang et al. (15) retrospectively found that the overall 1-year, 3-year, and 5-year OS of the RFA group was 73.9%, 51.1%, and 28.0%, respectively, while the TACE-RFA combined group was 88.5%, 64.6%, and 44.3%, respectively, and there were significant differences between the groups. In the latest retrospective multicenter study (16) of the First Affiliated Hospital of Sun Yat-sen University, 468 patients with single small HCC (≤3.0 cm) received RFA (322 cases) or TACE-RFA (146 cases), respectively, with 74.8% and 42.5% of the 1-year and 5-year OS rates in the combined group, and 53.5% and 28.7% in the RFA group (P<0.001), respectively. It has been determined that the combination of TACE-RFA can effectively reduce recurrence following RFA and improve the long-term survival rate of patients with small HCC. According to existing evidence-based medical literature, the cumulative 5-year survival rate of the TACE-RFA combination is superior, and the local control effect and long-term survival rate (> 5 years) are also more satisfactory. While combination therapy has effectively improved OS and prolonged recurrence-free survival (RFS), RFS was not assessed in this study due to the nature of early-stage HCC and its ‘on-off-on’ recurrence pattern (13). A phase III randomized controlled clinical study (14) found that the 5- and 7-year RFS of TACE-RFA and RFA groups were 41.4%, 34.5%, 27.4%, and 18.1%, respectively (HR=0.66, 95%CI: 0.49-0.89, P=0.007). A retrospective multicenter study (16) revealed that the RFS rates at 1 and 5 years were 51.7% and 24.4% in the combined group, and 36.1% and 9.3% in the RFA group (P<0.001). It is evident that the advantages of TACE-RFA over RFA monotherapy in RFS are undeniable.

**Figure 4:**
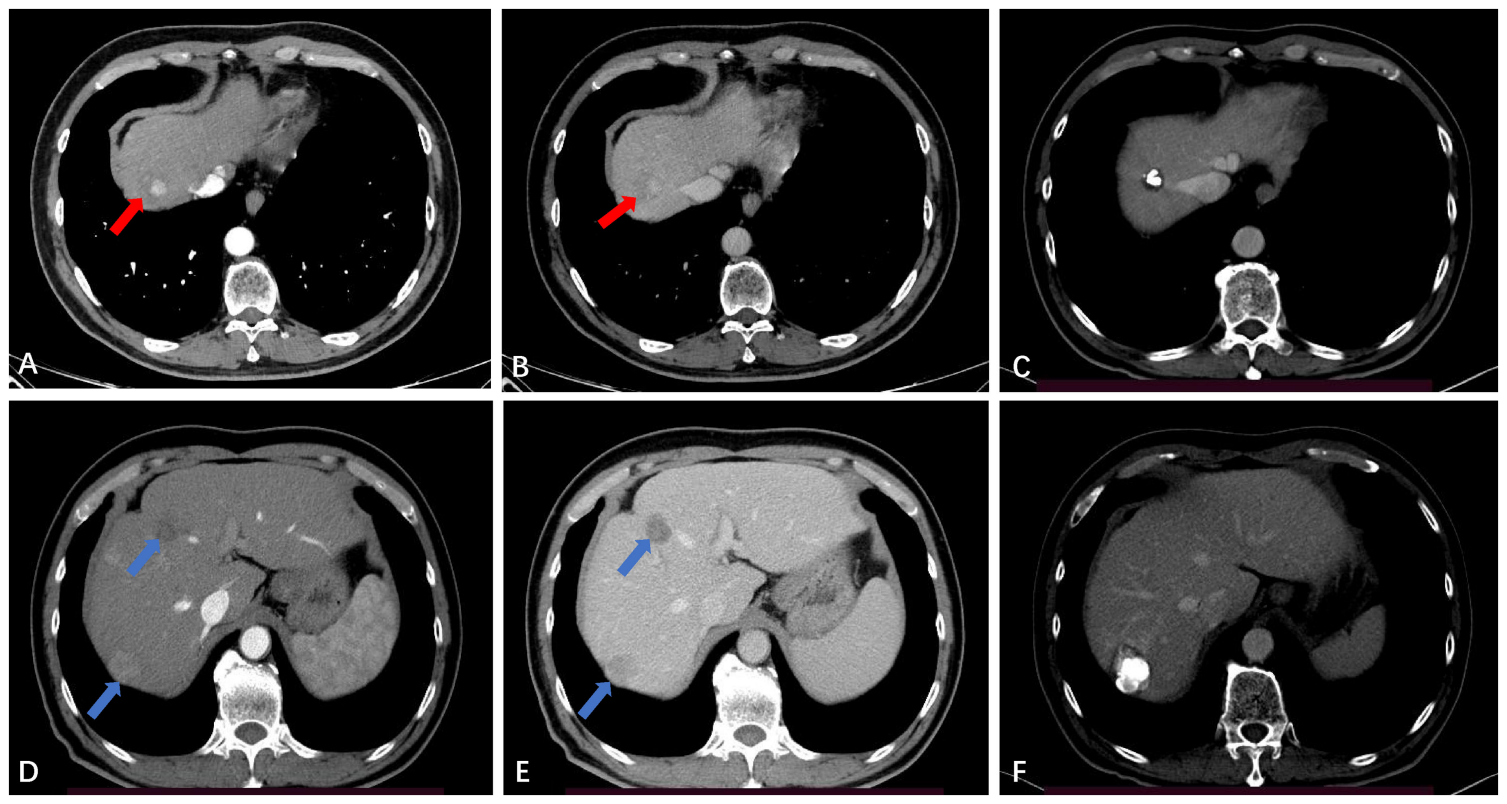
Intrahepatic distant recurrence (IDR) after TACE-RFA for HCC in a 56-year-old man. **(A, B)** Dynamic contrast-enhanced axial CT scan shows a single HCC with diameter of 2.4cm (red arrow). **(C)** Axial CT scan shows the complete ablation zone, with “intratumoral lipiodol deposition” visible in the ablation zone. **(D, E)** CT scan obtained during the hepatic arterial phase and portal venous phase 51 months after TACE-RFA shows IDR (blue arrows), with the largest lesions measuring about 2.7 cm. The two lesions received RFA and TACE-RFA, respectively. **(F)** Iodide oil deposits are visible in the largest lesion.

**Figure 5:**
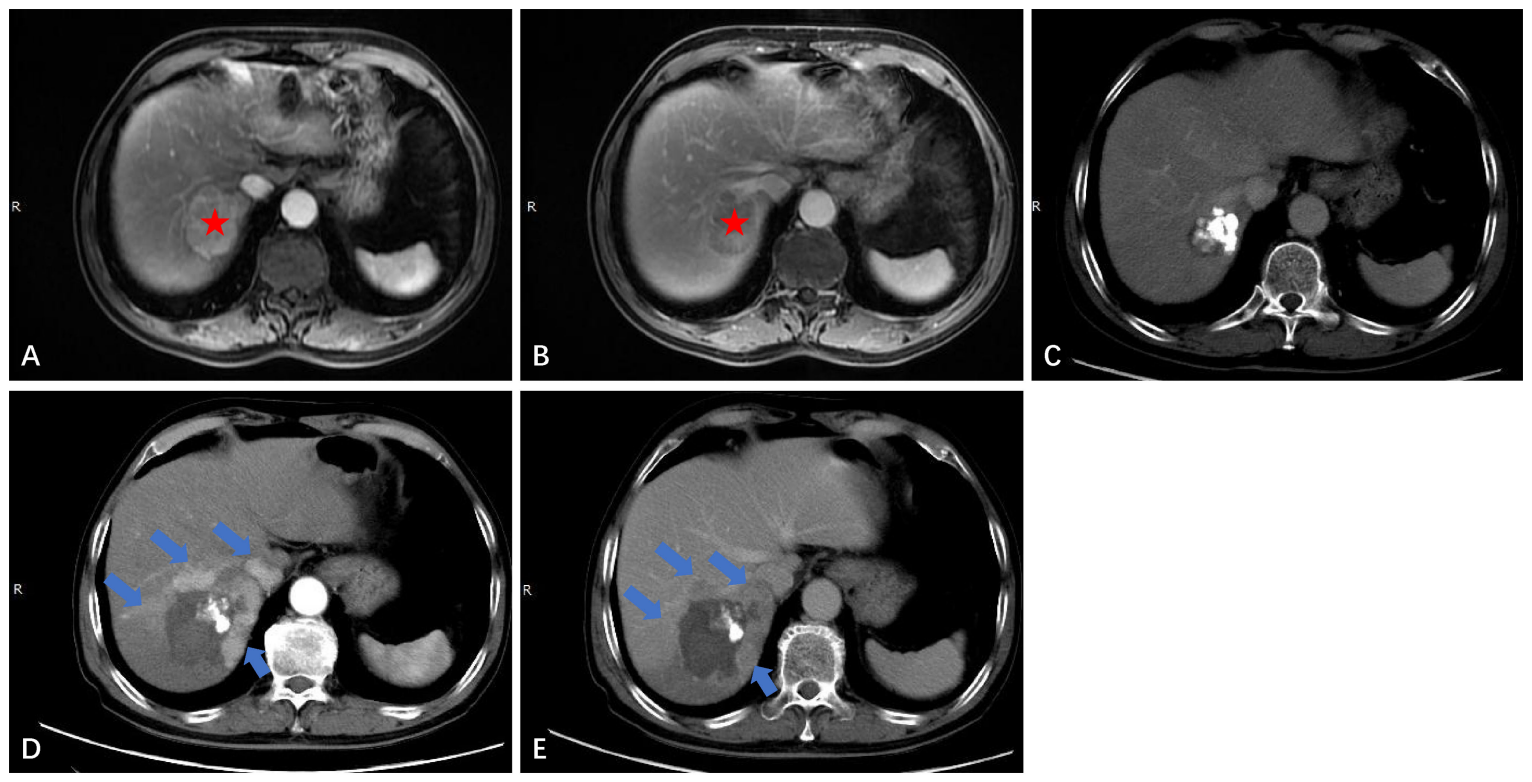
Local tumor progression (LTP) after TACE-RFA for HCC in a 72-year-old man. Finally, the patient received TACE as a second-line treatment. **(A)** Dynamic contrast-enhanced axial MRI scan shows a single 4.6cm×3.7cm×3.4cm HCC (red star). **(B)** MRI scan shows washout at the portal venous phase (red star). **(C)** CT scan obtained during the portal venous phase 1 month after TACE-RFA shows the complete ablation zone adjacent to the hepatic vein. **(D, E)** CT scan obtained during the hepatic arterial phase and portal venous phase 28 months after TACE-RFA shows the LTP, with multiple arterial enhancing nodules around the original ablation lesion and washout at the portal venous phase (blue arrows).

In this study, 66 out of 118 patients (55.9%) showed no recurrence or metastasis, 42 patients (35.6%) developed IDR (**Figure 4**), and only 3 patients (2.5%) experienced LTP during the entire follow-up period (**Figure 5**). The study results also revealed that the LTP rates at 1, 3, 5, and 7 years following TACE-RFA therapy were 0%, 4.6%, 4.6%, and 4.6%, respectively. These findings are consistent with previous studies (13, 17, 18) which also indicated LTP occurrences within the first 3 years, followed by a gradual decrease in the incidence rate. The study suggests that patients who undergo TACE-RFA therapy should have more frequent follow-up in the first 3 years after treatment. So, what factors are prone to LTP? A study by Huang et al. (19) recruited 249 patients into two groups (TACE group *vs* TACE-RFA group) based on diameter; they found that undergoing RFA alone and an insufficient ablative margin were independent risk factors for LTP. To minimize the adverse effects of these risk factors, we implemented a different approach compared to the study by Huang et al. (19). We directly treated single lesions ≤ 5 cm with TACE-RFA, which can reduce the problem of insufficient safety boundaries resulting from ablation alone. Additionally, conventional methods of ablation guidance such as US, CBCT, CT, and DSA can be enhanced by utilizing a multi-image guidance mode. This approach involves using US for puncture positioning and real-time monitoring of the ablation process, including observation of heat coverage and heat sink effect. CT or CBCT is used for the assessment of ablation areas and safety boundaries, with a multiple overlapping ablation strategy required for larger lesions (3-5 cm) to implement the entire ablation process. Following an ablation cycle, our center performs a CT or CBCT scan on the patient and reviews three-dimensional images in the coronal, sagittal, and axial positions using the post-processing workstation. This evaluation is done to determine if the angle, orientation, or umbrella needle should be adjusted. For lesions with obvious iodized oil deposits, DSA is also used to fluoroscopically adjust the ablation needles to ensure complete coverage of the entire tumor lesion. A previous study (20) found that imaging-invisible micrometastases may exist around HCC, and a 5-mm ablative margin is typically required to completely eradicate these microsatellite foci (12). However, in our center’s practice of expanding the safety ablation boundary to 1 cm, it is encouraging to note that the LTP was only 2.5% at the follow-up cut-off time in this study. Gui et al. reported 8.7% (21), and they conclusively showed that TACE + RFA provided comparable results compared to surgical resection. As with us, the effectiveness of combination therapy has been demonstrated, although the range of tumor diameters varies. The use of TACE in combination with RFA for HCC lesions presents several potential clinical implications. Firstly, this combination therapy may provide a curative treatment option for a subset of patients with HCC lesions that are not amenable to surgery or transplantation. Secondly, it may offer a potential bridge to liver transplantation by achieving tumor downstaging and preventing tumor progression in patients on the transplant waiting list. Additionally, the combination of TACE and RFA may contribute to improved OS and reduced recurrence rates in patients with larger HCC lesions, thus impacting long-term clinical outcomes. Further research and well-designed clinical trials are warranted to better define the role of this combined approach and to validate its potential benefits in improving patient outcomes. A retrospective study by Lee et al. (22) also suggested that the combination of TACE and RFA results in low LTP (about 5%). Previous studies by Cao et al. (13) showed that tumor size [hazard ratio (HR)=1.592, 95% CI: 1.313–1.930, P<0.001] is an independent risk factor for tumor progression. We speculate that this result may be due to most of the tumors in our included population being less than 3 cm in diameter. Therefore, we suggest that TACE-RFA should be considered for HCC ≤ 5 cm, with sufficiently large ablation boundaries being equally important. In addition to studying LTP, we observed recurrence patterns in 118 patients, with 42 (35.6%) having IDR, 4 (3.4%) experiencing extrahepatic recurrence, and 3 (2.5%) developing PVTT. A meta-analysis by Gui et al. (21) found that after undergoing TACE-RFA, 227 (43.0%) developed IDR, and 23 (3.7%) had extrahepatic recurrence, which aligns with our study.

In this study, we noted minor complications, but no major complications were observed. The minor complications improved with symptomatic treatment, suggesting that the treatment strategy is relatively safe and suitable for clinical promotion.

The reason our research achieved relatively satisfactory efficacy and safety is closely linked to the continuous improvement and maturity of current TACE or RFA treatments. More importantly, we utilized the TACE-RFA joint strategy to maximize its efficacy through: (a) providing additional survival benefit from chemoembolization; (b) using TACE before ablation to mark the lesion with iodine oil or microsphere deposition, enabling clear display of the lesion’s location under multi-image guidance combined with US/CT/CBCT/DSA and other imaging equipment, and assisting with positioning; (c) enhancing heat conduction through the deposition of iodine oil or microspheres in the tumor to better kill tumor cells and induce complete necrosis; (d) reducing the inherent “heat sink effect” of thermal ablation by effectively blocking the rich blood flow in and around the tumor lesion with TACE; (e) improving the prognosis of patients by detecting some microlesions as soon as possible through arteriography and early intervention; and (f) strictly adhering to safe ablation boundaries.

There were limitations to our study. Firstly, this is a retrospective, single-center study with limited sample sizes and inevitable selection bias. Secondly, this is a one-arm study that lacks head-to-head comparisons and can only compare efficacy and safety through historical controls. Thirdly, the time interval between TACE and RFA is typically within 4 weeks, so differences in the time interval between TACE and RFA may lead to biases in the evaluation of treatment efficacy and safety. However, there are few studies on the timing of treatment intervals for combination therapy, which is still in the exploratory stage, but most of them advocate ablation after TACE with an interval of 0-4 weeks (23–25). The main considerations are as follows: first, it takes time for patients to recover liver function after TACE. Second, excessively prolonging the time interval can lead to recanalization and neoangiogenesis of the cancer nest. However, some scholars think that RFA performed on the same day as chemoembolization has the greatest efficacy in tumor ablation (23). But the optimal interval between TACE and RFA remains a topic of debate, and individualized approaches based on tumor characteristics and patient-specific factors may be necessary. Further prospective studies are needed to clarify the optimal interval selection and to develop guidelines for the combined use of TACE and RFA in the treatment of HCC. In other words, the interval selection for TACE combined with RFA in the treatment of HCC is a complex and multifaceted issue that requires careful consideration of various clinical and tumor-specific factors. As research in this area continues to evolve, a better understanding of the optimal interval and its impact on treatment outcomes will further enhance the effectiveness of this combined therapeutic approach.

## Conclusion

In conclusion, our study found that TACE-assisted multi-image-guided RFA is a feasible and safe treatment option for stage Ia HCC of CNLC. The combination therapy of TACE-RFA should be considered as one of the modified ablation strategies that provide a more effective treatment for stage Ia HCC of CNLC.

## Data availability statement

The original contributions presented in the study are included in the article/supplementary material. Further inquiries can be directed to the corresponding authors.

## Ethics statement

The studies involving humans were approved by the Institutional Review Boards of the Peking University First Hospital. The studies were conducted in accordance with the local legislation and institutional requirements. The ethics committee/institutional review board waived the requirement of written informed consent for participation from the participants or the participants’ legal guardians/next of kin because this was a retrospective study.

## Author contributions

YX: Data curation, Formal analysis, Resources, Software, Writing – original draft. TL: Data curation, Formal analysis, Resources, Software, Writing – original draft. LS: Investigation, Methodology, Writing – review & editing. XT: Investigation, Methodology, Writing – review & editing. JW: Conceptualization, Supervision, Writing – review & editing. YZ: Conceptualization, Supervision, Writing – review & editing.
